# Impact of early β-blocker use on the incidence of sepsis and clinical outcomes following cardiac surgery: a retrospective cohort study

**DOI:** 10.3389/fphar.2025.1615868

**Published:** 2025-07-30

**Authors:** Chen Yin, Chengjian Guan, Qianli Ma, Shaotong Zhang, Qian Chen, Bing Xiao

**Affiliations:** ^1^ Department of Cardiac Surgery, The Second Hospital of Hebei Medical University, Shijiazhuang, China; ^2^ Department of Cardiology, The Second Hospital of Hebei Medical University, Shijiazhuang, China; ^3^ Department of Physiology, Hebei Medical University, Shijiazhuang, China

**Keywords:** sepsis, cardiac surgery, perioperative management, MIMIC-IV database, postoperative outcomes

## Abstract

**Background:**

Sepsis after cardiac surgery represents a severe perioperative complication with high incidence and mortality rates. While the cardioprotective benefits of β-blocker following cardiac surgery are widely recognized, their impact on sepsis development remains unclear. This study aims to investigate the association between early postoperative β-blocker use and the incidence of sepsis, as well as clinical outcomes, in patients undergoing cardiac surgery.

**Methods:**

The analysis incorporated data from the MIMIC-IV database, with confounding factors addressed through propensity score matching (PSM), inverse probability of treatment weighting (IPTW), and overlap weighting (OW). Logistic regression models assessed the risk of sepsis and in-hospital mortality, while Cox proportional hazards models evaluated 28-day and 1-year mortality. Kaplan-Meier survival curves and log-rank tests compared survival between groups. Sensitivity analyses using Fine-Gray competing risk models and cumulative incidence functions were performed. Subgroup analyses explored heterogeneity of treatment effects, and metoprolol was further stratified by dose to assess dose-response relationships.

**Results:**

A total of 3,154 patients treated with β-blocker and 5,220 controls were included. Early β-blocker use was associated with a reduced risk of sepsis and lower in-hospital mortality across all methods. For 28-day and 1-year mortality, β-blocker use showed a trend toward risk reduction. Competing risk analyses demonstrated lower cumulative incidence of sepsis in the β-blocker group. Subgroup and dose-response analyses indicated that both low and high doses of metoprolol were associated with reduced postoperative sepsis risk and mortality outcomes.

**Conclusion:**

Early use of β-blocker after cardiac surgery was associated with a lower incidence of sepsis, with potential benefits observed in both short-term and long-term prognosis. These findings provide valuable evidence for optimizing perioperative drug management strategies.

## 1 Introduction

Postoperative sepsis is one of the severe complications in the perioperative period following cardiac surgery (Howitt et al., 2018; Lagrost et al., 2014). A retrospective study based on the Sepsis-3 diagnostic criteria revealed that, among cardiac surgery patients admitted to the intensive care unit (ICU), both suspected and confirmed sepsis patients had significantly longer ICU stays and higher mortality rates compared to those without evidence of infection. Specifically, the 30-day mortality rate among confirmed sepsis patients was increased by 6.6-fold (Howitt et al., 2018). Due to prolonged surgical procedures and the use of cardiopulmonary bypass (CPB), patients undergoing cardiac surgery were more prone to inflammatory responses and organ dysfunction (Squiccimarro et al., 2019; Paparella et al., 2002). Despite a range of perioperative management strategies currently in use (Whitlock et al., 2015; Goetz et al., 2021), and multiple prediction models for adverse events after cardiac surgery have been developed (Luo, 2024), no specific pharmacological interventions have been developed to target sepsis related to cardiac surgery. Given the poor prognosis caused by sepsis (Peng et al., 2024; Martin-Loeches et al., 2024), it is particularly urgent to explore effective drug treatment regimens.

Surgical stress-induced sympathetic activation has been recognized as a key mechanism affecting immune status in these patients. During the perioperative period, activation of the hypothalamus-pituitary-adrenal (HPA) axis and the sympathoadrenal system (SAS) promotes the release of cortisol and catecholamines (Marik and Flemmer, 2012). Since multiple immune cells express adrenergic receptors on their surfaces (Schiller et al., 2021; Lorton and Bellinger, 2015), these stress hormones engage receptor-mediated signaling pathways that disrupt the balance of immune cell quantity and function. Moreover, they stimulate immune cells to produce and release both pro-inflammatory and anti-inflammatory mediators, thereby increasing susceptibility to infection (Marik and Flemmer, 2012; Wang et al., 2005).

β-blocker, commonly used as part of perioperative care medications after cardiac surgery, have been well-established for their cardioprotective effects (Bello et al., 2023; Lindgren et al., 2022). Emerging evidence suggests that β-blocker may also play a significant role in modulating immune responses. Previous studies have indicated that septic patients receiving chronic β-blockers therapy tend to experience relatively less severe disease conditions (Hasegawa et al., 2022; Tan K. et al., 2021). Furthermore, an animal experiment has confirmed that intraoperative administration of esmolol can attenuate the systemic inflammatory response after pneumonectomy (Garutti et al., 2019). However, there is currently a lack of cohort study investigating whether early postoperative β-blocker use can reduce the incidence of sepsis after cardiac surgery.

Therefore, we conducted a retrospective cohort study to examine the association between early postoperative β-blockers use and sepsis development in cardiac surgery patients, aiming to provide insights for optimizing perioperative pharmacological strategies to prevent this serious complication.

## 2 Materials and methods

### 2.1 Data sources and study design

This study utilized data from the Medical Information Mart for Intensive Care-IV (MIMIC-IV, version 3.1) (Johnson et al., 2023), encompassing patient information collected at Beth Israel Deaconess Medical Center spanning from 2008 to 2022, including 94,458 ICU admissions. The database was stored in PhysioNet (https://physionet.org/) (Goldberger et al., 2000); due to its strict de-privacisation procedures, patient informed consent was not required. The corresponding author obtained the necessary database access by completing the Collaborative Institutional Training Initiative (CITI) program exam (Record ID: 57440109). Patients meeting all of the following criteria were eligible for inclusion (Supplementary Figure S1): having undergone cardiac surgery (including valve surgery, coronary artery bypass grafting, or combined procedures) during their hospital stay and being over 18 years of age. For patients with multiple ICU admissions, only the first admission was considered. We extracted comprehensive clinical variables from the first day of ICU admission. Baseline characteristics included gender, race, age, body mass index (BMI). Past medical history comprised myocardial infarction (MI), hypertension, atrial fibrillation (AF), kidney disease, lung disease, liver disease, diabetes, malignancy and rheumatic disease. Laboratory test indicators encompassed white blood cell (WBC), red blood cell (RBC), platelet, hemoglobin, red cell distribution width (RDW), hematocrit, international normalized ratio (INR), prothrombin time (PT), partial thromboplastin time (PTT), anion gap, bicarbonate, blood urea nitrogen (BUN), creatinine, glucose, sodium, calcium, potassium, magnesium, chloride, N-terminal pro-B-type natriuretic peptide (NT-proBNP), C-reactive protein (CRP), albumin, alanine aminotransferase (ALT), aspartate aminotransferase (AST), alkaline phosphatase (ALP), total bilirubin, basophils, eosinophils, lymphocytes, monocytes, neutrophils, lactic acid and urine output. Vital signs consisted of oxygen partial pressure (PO_2_), carbon dioxide partial pressure (PCO_2_), PH, total carbon dioxide (Total CO_2_), heart rate (HR), systolic blood pressure (SBP), diastolic blood pressure (DBP), mean arterial pressure (MBP), central venous pressure (CVP), respiratory rate (RR), percutaneous arterial oxygen saturation (SpO_2_) and temperature. Therapeutic interventions were recorded, including the administration of vasoactive drugs, angiotensin converting enzyme inhibitors (ACEI), glucocorticoid, anticoagulant, antibiotics, diuretics, and β-blocker, as well as the implementation of renal replacement therapy (RRT) and mechanical ventilation. The Sequential Organ Failure Assessment (SOFA) score, Charlson Comorbidity Index, Simplified Acute Physiology Score III (Apsiii) and Oxford acute severity of illness score (Oasis) were also obtained on the first ICU day. For parameters with multiple measurements, the first recorded value was used for analysis. Early β-blocker use was defined as prescription within the first day after cardiac surgery. Patients who received prescriptions after the diagnosis of sepsis were excluded. The primary endpoint was the incidence of sepsis in the ICU, diagnosed according to the Sepsis-3 criteria. The secondary endpoints included in-hospital mortality, 28-day mortality, and 1-year mortality.

### 2.2 Statistical analysis

To address potential confounding factors, we employed propensity score matching (PSM), inverse probability of treatment weighting (IPTW) and overlap weighting (OW) methods. For PSM, the MatchIt package (version 4.7.0) was used to perform 1:1 nearest neighbor matching with a caliper width of 0.2 standard deviations. The IPTW approach involved calculating propensity scores via logistic regression, which were then used to assign weights to patients for subsequent analyses. The OW emphasized the population where treatment groups overlapped, with weights proportional to the probability of assignment to the opposite treatment group, thereby automatically eliminating extreme weights. Continuous variables’ distribution was evaluated using the Kolmogorov-Smirnov test, with normally distributed variables expressed as mean ± standard deviation (SD) and non-normally distributed variables as median (interquartile range). Kruskal–Wallis tests were used for between-group comparisons of continuous variables, while Pearson’s chi-square tests were applied to categorical variables expressed as frequencies (proportions). We assumed that the data were Missing at Random (MAR), calculated the missingness ratio for each variable, and removed those with a missing rate exceeding 20% (Supplementary Figure S2). Data preprocessing included handling extreme outliers based on the 1.5 times interquartile range rule and handling missing data through multiple imputations (10 imputations) using chained equations (MICE). Logistic regression was employed to assess the association between early β-blocker use and in-hospital sepsis and mortality, while Cox proportional hazards models were used to evaluate 28-day and 1-year mortality outcomes. Kaplan-Meier curves were utilized to observe survival patterns, with comparisons made using the log-rank test. Additionally, prespecified subgroup analyses were performed to examine heterogeneity of treatment effects across different populations. Regression analysis included all matching variables to ensure the robustness of the results. The competitive risk of sepsis occurrence was evaluated using a competitive risk model, specifically the Fine-Gray model. Patients who received metoprolol treatment were screened and retained for analysis, and the association between different doses and clinical outcomes was examined. All statistical analyses were carried out using R software (version 4.3.3), with statistical significance set at a two-sided *P*-value <0.05.

## 3 Results

**FIGURE 1:**
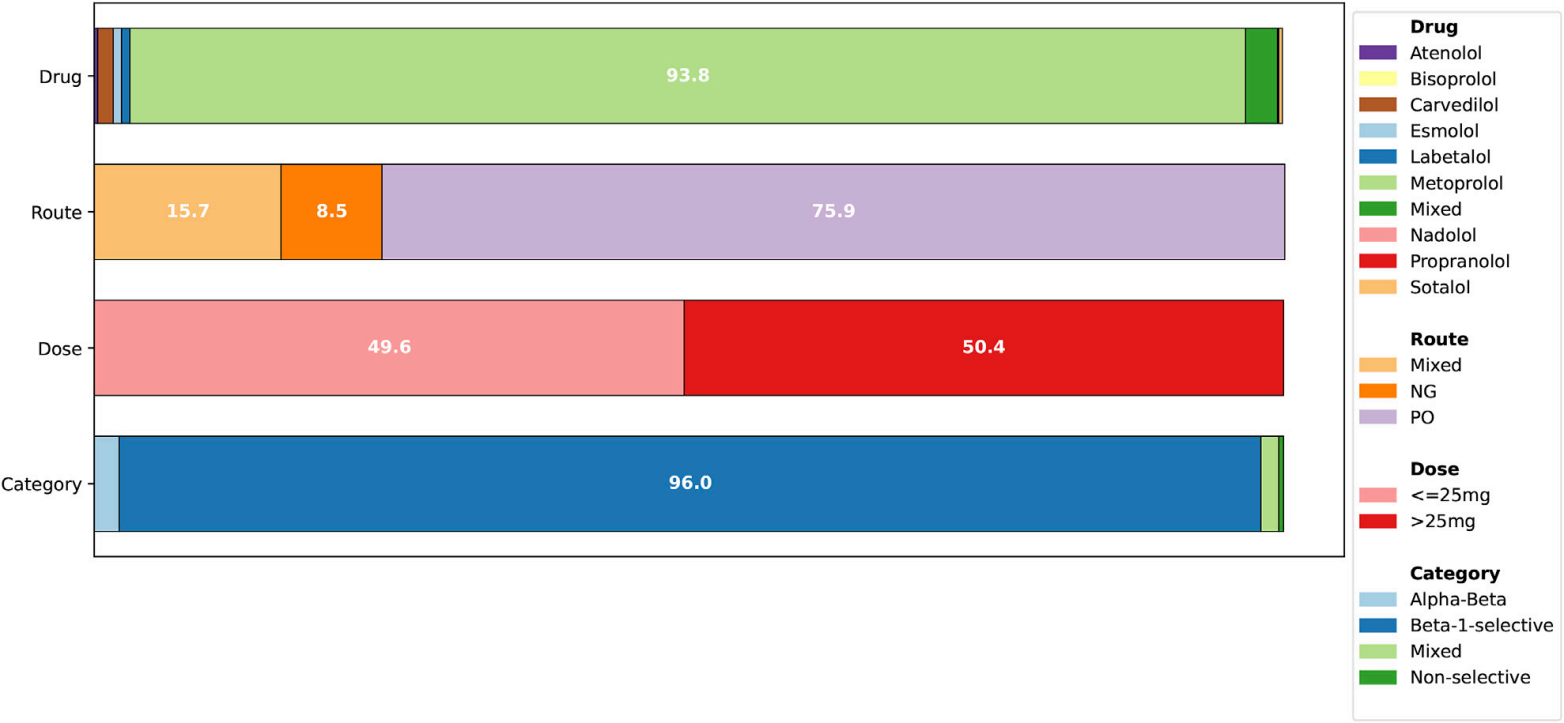
Distribution of early β-blocker.

This study included 3,154 patients treated with β-blocker and 5,220 control patients. Significant differences were observed in baseline characteristics between the two groups (Supplementary Table S1). From the perspective of demographic characteristics, the β-blocker group had a higher proportion of males, younger age, and greater BMI compared to the control group. Regarding laboratory parameters, the β-blocker group exhibited lower levels of WBC, RDW, BUN, chloride, magnesium, sodium, and creatinine, as well as improved coagulation function (lower INR, PT, and PTT) compared to the control group. Conversely, the β-blocker group had higher levels of RBC, hemoglobin, hematocrit. Hemodynamic parameters revealed that the β-blocker group had lower HR, higher SBP, DBP, and MBP compared to controls. In terms of comorbidities and complications, the β-blocker group presented with lower Charlson comorbidity index, SOFA, Apsiii, Oasis, and a lower prevalence of AF, chronic pulmonary disease, chronic kidney disease, chronic liver disease, and sepsis. However, this group had a higher proportion of patients with MI, hypertension and diabetes. The β-blocker group also had greater use of diuretics and ACEI but a lower requirement for mechanical ventilation and vasoactive drugs. Analysis of clinical outcomes indicated that the β-blocker group experienced lower in-hospital, 28-day, and 1-year mortality, accompanied by shorter hospital and ICU stays compared to the control group. Standardized mean difference (SMD) analysis suggested that most of these differences were clinically relevant (SMD >0.1). Figure 1 showed the application characteristics of β-blocker. The majority of patients received β-1 blockers (96%), with metoprolol being the primary agent (93.8%). Oral administration (PO) was the predominant route (75.9%), while intravenous (IV) and mixed routes were used less frequently. The distribution of dosing was nearly balanced, with 49.6% of patients receiving a low dose (≤25 mg) and 50.8% receiving a high dose (>25 mg).

**FIGURE 2:**
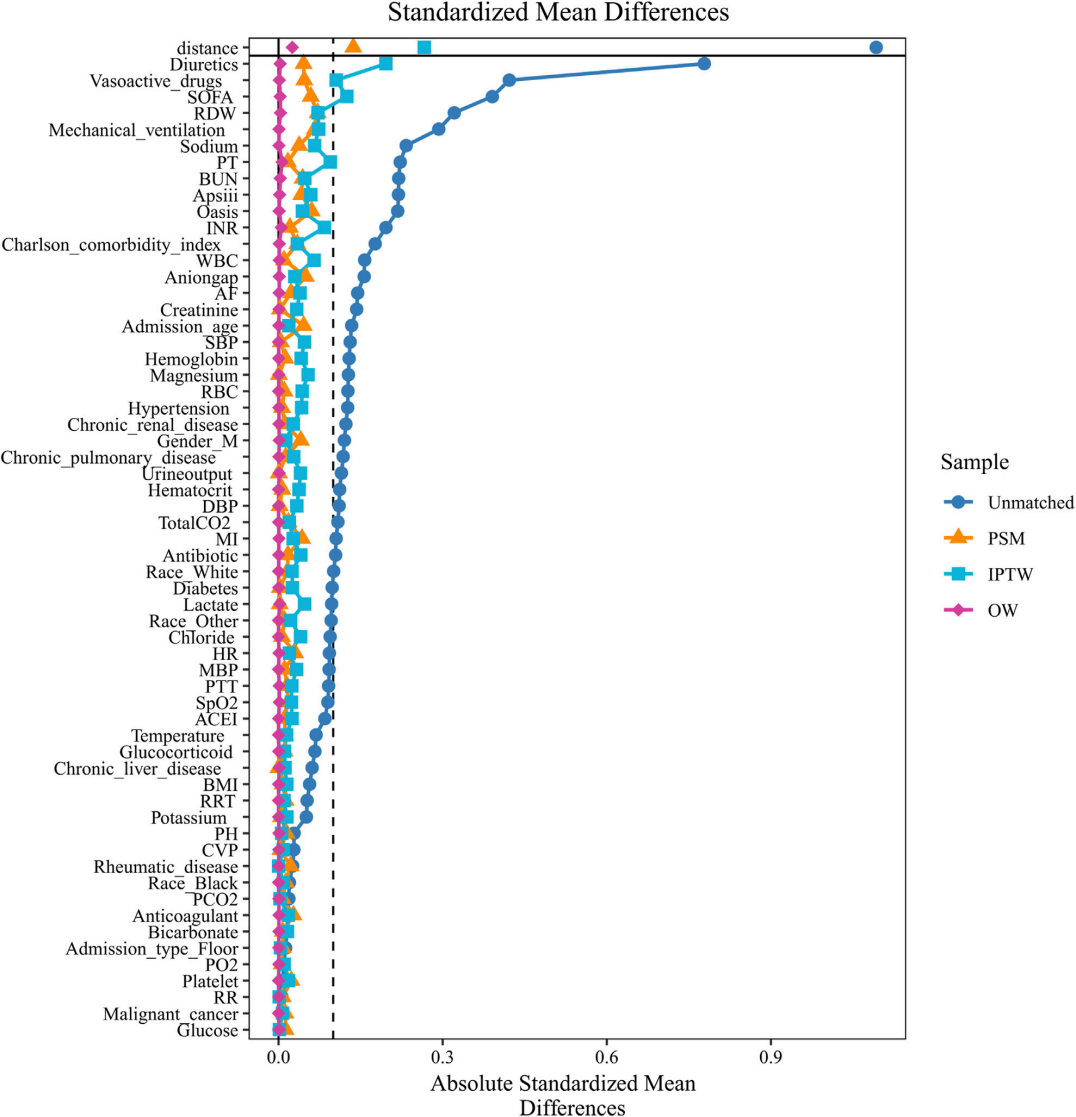
Covariate balance assessment across different matching methods. BMI, body mass index; WBC, white blood cell; RBC, red blood cell; RDW, red cell distribution width; INR, international normalized ratio; PT, prothrombin time; PTT, partial thromboplastin time; BUN, blood urea nitrogen; PO2, oxygen partial pressure; PCO2, carbon dioxide partial pressure; Total CO2, total carbon dioxide; HR, heart rate; SBP, systolic blood pressure; DBP, diastolic blood pressure; MBP, mean blood pressure; RR, respiratory rate; SpO2, percutaneous arterial oxygen saturation; SOFA, Sequential organ failure score; Apsiii, Simplified Acute Physiology Score III; Oasis, Oxford acute severity of illness score; MI, myocardial infarction; AF, atrial fibrillation; RRT, renal replacement therapy.

To assess the balancing effects of PSM, IPTW, and OW, we conducted evaluations by comparing changes in SMD before and after adjustment (Figure 2; Supplementary Tables S2–S4). The results revealed baseline imbalances between the β-blocker group and the control group in the original data, with most variables exhibiting SMD values exceeding the 0.1 threshold. However, after adjustment using PSM, IPTW, and OW methods, the SMD values for most of variables were reduced to below 0.1.

**FIGURE 3:**
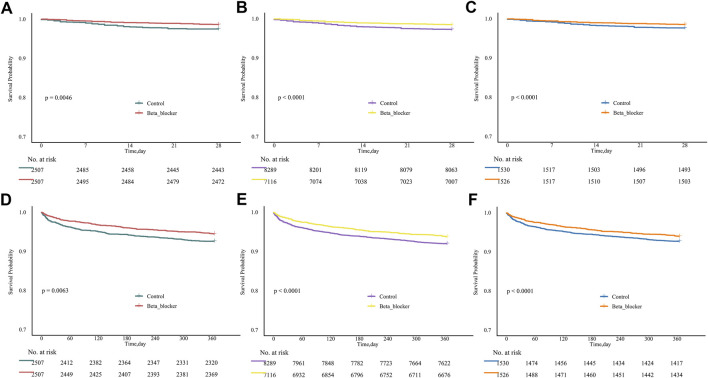
Kaplan-Meier survival curves using different propensity score methods. **(A–C)** 28-day survival curves using PSM, IPTW, and OW methods, respectively. **(D–F)** 1-year survival curves using PSM, IPTW, and OW methods, respectively. PSM, propensity score matching; IPTW, inverse probability of treatment weighting; OW, overlap weighting.

The Kaplan-Meier survival analysis within the PSM cohort demonstrated that the 28-day survival rate was significantly higher in the β-blocker group compared to the control group (Figure 3A, *P* = 0.0046). This survival benefit persisted throughout the entire 1-year follow-up period (Figure 3D, *P* = 0.0063). These findings were further corroborated by IPTW analysis, which showed significantly higher survival rates in the β-blocker group at both 28 days (Figure 3B, *P* < 0.001) and 1 year (Figure 3E, *P* < 0.001). Similarly, overlap weighting analysis consistently confirmed the survival advantage for the β-blocker group at both 28-day (Figure 3C, *P* < 0.001) and 1-year mortality (Figure 3F, *P* < 0.001).

The clinical benefits were consistently demonstrated in multivariable-adjusted analyses (Table 1). All three models showed a marked reduction in sepsis risk with β-blocker treatment (pooled OR ≈ 0.13, all *P* < 0.001). Likewise, β-blocker use correlated with decreased in-hospital mortality (OR range: 0.455–0.506, *P* = 0.002–0.04). A similar protective effect was observed for 28-day mortality (HR range: 0.598–0.808, all *P* < 0.05), and this survival benefit extended to 1-year mortality (HR range: 0.784–0.806, all *P* < 0.05).

**TABLE 1:** Clinical outcomes across different propensity score methods.

Methods	Sepsis		Hospital_death		28 days_death		1 year_death	
	OR (95% CI)	P	OR (95% CI)	P	HR (95% CI)	P	HR (95% CI)	P
PSM	0.175 (0.150–0.203)	<0.001	0.455 (0.260–0.774)	0.04	0.598 (0.386–0.925)	0.021	0.784 (0.624–0.984)	0.036
IPTW	0.176 (0.154–0.202)	<0.001	0.497 (0.318–0.777)	0.002	0.640 (0.436–0.939)	0.023	0.806 (0.656–0.990)	0.04
OW	0.179 (0.156–0.205)	<0.001	0.506 (0.323–0.794)	0.003	0.637 (0.432–0.941)	0.023	0.788 (0.644–0.965)	0.021

OR, odds ratio; HR, hazard ratio; PSM, propensity score matching; IPTW, inverse probability of treatment weighting; OW, overlap weighting.

**FIGURE 4:**
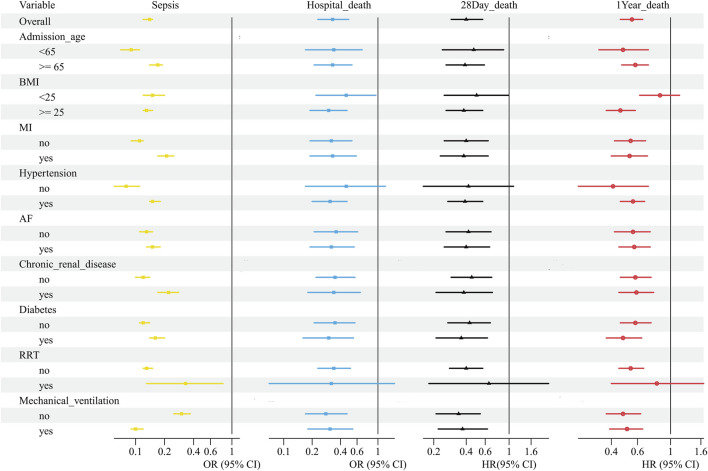
Subgroup analysis of clinical outcomes associated with β-blocker treatment. MI, myocardial infarction; AF, atrial fibrillation; RRT, renal replacement therapy; OR, odds ratio; HR, hazard ratio.

To evaluate the heterogeneity of β-blocker treatment effects across various subgroups, a prespecified subgroup analysis was performed (Figure 4). The results indicated that the treatment effects of β-blocker remained consistent across most subgroups. We conducted a sensitivity analysis to examine the association between β-blocker use and postoperative sepsis using a competitive risk model. Fine-Gray subquantile risk regression was applied, treating in-hospital deaths as competing events, to analyze the impact of β-blocker use on sepsis risk. The results showed that the cumulative incidence of sepsis in the β-blocker use group was significantly lower than that in the control group (Supplementary Figure S4). In the competitive risk regression analysis, the adjusted subdistribution hazard ratio (SHR) for β-blocker use was 0.25 (95% CI: 0.22–0.28), suggesting an association between β-blocker use and reduced sepsis risk. Supplementary Figure S5 presented the dose-specific subgroup analyses. Both low and high doses of metoprolol were associated with lower postoperative sepsis compared with nonusers. This pattern was consistent across all measured outcomes, including in-hospital mortality, 28-day mortality, and 365-day mortality, where both dosing regimens showed protective effects but the magnitude of benefit was generally bigger in the low-dose group.

## 4 Discussion

We conducted a retrospective study to investigate the impact of early postoperative β-blocker use on the occurrence and prognosis of sepsis. Our results revealed that early administration of beta-blockers was associated with a reduced incidence of sepsis after cardiac surgery, and improved inpatient mortality, 28-day mortality, and 1-year mortality rates. These associations remained robust after adjusting for confounding factors using three statistical methods, including PSM, IPTW, and OW. Moreover, subgroup analysis indicated that the beneficial effects of β-blocker were consistent across most patient populations. Sensitivity analyses using competing risk models confirmed the robustness of these associations. Dose-response analysis showed that both low and high doses of metoprolol were associated with reduced sepsis risk, with low-dose groups also demonstrating favorable trends in mortality outcomes. These findings suggest that early β-blocker therapy might be an effective strategy to reduce the risk of sepsis and improve prognosis during the perioperative period following cardiac surgery.

Despite some advancements in reducing systemic inflammatory responses, such as improvements in CPB equipment, ultrafiltration/hemofiltration, and cytokine hemadsorption techniques, CPB-associated tissue/organ dysfunction and inflammatory complications, remain inadequately addressed (Mangoush et al., 2007; Dekker et al., 2020; Journois et al., 1994; Becker et al., 2023). Surgical site infections continue to pose a significant challenge, even with the implementation of various preventive measures targeting infection and bleeding control (Pradeep et al., 2021). Given the high mortality rate associated with sepsis (Rudd et al., 2020), identifying effective interventions to comprehensively address these critical postoperative complications is imperative.

As mentioned above, cardiopulmonary bypass (CPB) during cardiac surgery is a major factor inducing systemic inflammatory response syndrome (SIRS). Blood contact with the artificial surfaces of the extracorporeal circuit may activate multiple pro-inflammatory cascade reactions, thereby increasing the risk of infection and organ dysfunction (Day and Taylor, 2005; Suleiman et al., 2008). In recent years, studies have found that β-blocker, beyond their traditional cardioprotective effects, possess potential anti-inflammatory properties. Wong et al. reported that carvedilol (CVL) can prevent lysosomal and mitochondrial dysfunction, reduce ASC oligomerization, induce autophagy through a SIRT1-dependent pathway, and inhibit the activation of the NLRP3 inflammasome (Wong et al., 2018). Furthermore, carvedilol has been shown to inhibit oxidative stress and apoptosis by activating the Nrf2/ARE signaling pathway (Zhang et al., 2022). Another critical factor was the elevation of endotoxin levels during CPB, which contributed to increased postoperative infection risk (Kats et al., 2011; Klein et al., 2011). Studies have shown that patients undergoing CPB experience inadequate intestinal perfusion, leading to a significant increase in intestinal permeability that can persist beyond 24 h postoperatively (Rocke et al., 1987; Riddington et al., 1996). Early research confirmed this phenomenon by monitoring dynamic changes in lipopolysaccharide concentrations in venous blood of CPB patients (Ohri et al., 1993). As a key inflammatory trigger, endotoxin can activate both humoral and cellular mediator systems, initiating a cytokine cascade reaction typically characterized by the early release of TNF-α, which subsequently induces the expression of pro-inflammatory cytokines such as IL-6 and IL-8. These endotoxin-induced inflammatory responses elicit systemic compensatory mechanisms including neuroendocrine responses (Lowry, 2005). Notably, recent research has found that β-1 blocker can attenuate both systemic and local inflammatory responses in open abdominal surgery via a cholecystokinin receptor-dependent mechanism, while maintaining the integrity of intestinal barrier function (Tan S. et al., 2021). Furthermore, stress responses trigger a surge in catecholamines release, which binds to beta-adrenergic receptors (β-AR) on cell surfaces, activating downstream signaling pathways that adversely affect soft tissue wound healing processes (Jia et al., 2024). These effects include prolonged neutrophil retention at the wound site, excessive secretion of inflammatory factors, inhibition of ERK phosphorylation impeding keratinocyte migration, suppression of angiogenesis, altered fibroblast function, and upregulated expression of matrix metalloproteinases (MMPs) (Dasu et al., 2014; Yang et al., 2021; Kruk et al., 2019; Chay et al., 2016). This series of cascade reactions ultimately impairs the wound healing process, suggesting that β-blocker may exert a positive influence on wound healing.

Although we applied a competing risk model (Fine-Gray model) to account for competing effects such as death, the positive effect size for β-blocker use in sepsis risk reduction remained low (SHR: 0.25, 95% CI: 0.22–0.28). This implied that competing risk alone might not fully explain the weak association. While few studies have specifically examined β-blockers’ impact on postoperative sepsis risk, existing evidence indicates that early β-blocker use might exert anti-inflammatory effects by reducing circulating inflammatory proteins and suppressing pro-inflammatory cytokines like IL-6 (El-Menyar et al., 2024; Ogawa et al., 2013; Sezai et al., 2012). Unfortunately, the absence of key inflammatory biomarkers, including IL-6 and CRP, in our database limited further exploration of potential associations between cytokine levels and β-blocker use in this patient cohort. In additional, the analysis might be confounded by selection bias, as patients receiving β-blocker typically have more favorable baseline prognoses. Retrospective diagnosis could further distort findings through false-positive cases, particularly among cardiac surgery patients receiving antibiotics or blood cultures. Therefore, although sensitivity analyses including competing risk models yielded consistent results, the observed association and its effect size require cautious interpretation. Prospective studies remain necessary to establish whether β-blocker genuinely protect against postoperative sepsis and to determine their clinical utility.

In evaluating in-hospital mortality and long-term prognosis, our study demonstrated that early β-blocker administration was significantly associated with a reduction in both short-term and long-term mortality. These findings were robust across multiple analytical approaches highlighting the potential clinical value of β-blocker in the postoperative management of patients at risk for sepsis. While current studies lack full consensus, exposure to β-blocker prior to sepsis onset has shown prognostic benefits (Fuchs et al., 2017), and accumulating evidences support their therapeutic application during sepsis to improve clinical outcomes (Tan K. et al., 2021; Kuo et al., 2021). Therefore, the potential benefits of early postoperative β-blocker use deserve further validation through prospective studies in the future.

Dose-response analysis revealed that both low and high doses of β-blocker were associated with a reduction in sepsis risk; however, the magnitude of benefit was more pronounced in the low-dose group. These findings suggest that lower doses of metoprolol may be sufficient to confer clinical benefit, and escalating the dose may not yield additional protective effects. This observation was consistent with previous studies in other clinical settings, such as acute myocardial infarction, where higher doses of β-blockers have not demonstrated superior outcomes compared to lower doses (Allen et al., 2017; Hwang et al., 2019). Furthermore, current guidelines advise against the routine use of high-dose β-blockers during the perioperative period of non-cardiac surgery (Smilowitz et al., 2020). Therefore, rational selection of β-blocker dose is crucial for optimizing patient prognosis.

Several limitations to this study should be acknowledged. Firstly, the retrospective nature of the study using the MIMIC-IV database introduces the possibility of selection bias and the influence of unknown confounding factors. Although we employed multiple statistical techniques to mitigate confounding, residual biases cannot be fully excluded. Secondly, the relative effects and duration of specific β-blocker classes need further study. Meanwhile, future research should incorporate a series of measurements to better capture the dynamic changes of patients. Finally, prospective randomized controlled trials are necessary to validate these findings and to confirm their generalizability to broader patient populations. Caution is advised when extrapolating our results beyond the studied cohort.

## 5 Conclusion

Early use of β-blocker after cardiac surgery was associated with a lower incidence of sepsis, and potential benefits were shown in both short-term and long-term prognosis. These findings were consistently validated across multiple statistical methods, providing valuable evidence to guide the optimization of perioperative drug management strategies.

## Data Availability

The original contributions presented in the study are included in the article/Supplementary Material, further inquiries can be directed to the corresponding author.
