# Supplementary material for: Impact of early β-blocker use on the incidence of sepsis and clinical outcomes following cardiac surgery: a retrospective cohort study
Source: Front Pharmacol. 2025 Jul 30;16:1615868. doi: 10.3389/fphar.2025.1615868 (PMC12343738; doi:10.3389/fphar.2025.1615868)
Supplement: Supplementary file 1 [file Supplementaryfile1.docx]

Supplementary Material

# Supplementary Figures and Tables

## Supplementary Tables

Supplementary Table 1. Baseline characteristics of unmatched patients

| Variables | Control (N = 5220) | β-blocker (N = 3154) | *P* | SMD |
| --- | --- | --- | --- | --- |
| Gender (%) |  |  | <0.001 | 0.117 |
| Female | 1661 (31.8) | 836 (26.5) |  |  |
| Male | 3559 (68.2) | 2318 (73.5) |  |  |
| Admission_type (%) |  |  | 0.588 | 0.013 |
| Emergency | 2249 (43.1) | 1339 (42.5) |  |  |
| Floor | 2971 (56.9) | 1815 (57.5) |  |  |
| Race (%) |  |  | <0.001 | 0.104 |
| Black | 209 (4.0) | 139 (4.4) |  |  |
| Other | 1160 (22.2) | 835 (26.5) |  |  |
| White | 3851 (73.8) | 2180 (69.1) |  |  |
| Admission_age | 69.50 (12.17) | 68.01 (11.18) | <0.001 | 0.128 |
| BMI | 29.50 (6.16) | 29.83 (5.75) | 0.016 | 0.055 |
| WBC | 12.34 (6.89) | 11.55 (5.04) | <0.001 | 0.131 |
| RBC | 3.26 (0.71) | 3.35 (0.68) | <0.001 | 0.125 |
| Platelet | 162.24 (68.49) | 161.76 (65.58) | 0.753 | 0.007 |
| Hemoglobin | 9.82 (2.08) | 10.08 (2.01) | <0.001 | 0.127 |
| RDW | 14.17 (1.80) | 13.67 (1.55) | <0.001 | 0.296 |
| Hematocrit | 29.70 (6.12) | 30.35 (5.82) | <0.001 | 0.109 |
| INR | 1.45 (0.46) | 1.40 (0.30) | <0.001 | 0.153 |
| PT | 15.98 (5.62) | 15.26 (3.25) | <0.001 | 0.157 |
| PTT | 41.28 (25.31) | 39.14 (23.28) | <0.001 | 0.088 |
| Aniongap | 11.92 (3.35) | 11.41 (3.22) | <0.001 | 0.153 |
| Bicarbonate | 23.15 (2.99) | 23.19 (2.65) | 0.527 | 0.014 |
| BUN | 20.55 (12.95) | 18.33 (10.10) | <0.001 | 0.191 |
| Creatinine | 1.17 (1.15) | 1.04 (0.87) | <0.001 | 0.122 |
| Glucose | 126.79 (41.17) | 126.74 (52.13) | 0.96 | 0.001 |
| Sodium | 138.70 (3.31) | 138.01 (2.95) | <0.001 | 0.22 |
| Potassium | 4.30 (0.58) | 4.33 (0.51) | 0.039 | 0.047 |
| Magnesium | 2.41 (0.57) | 2.34 (0.50) | <0.001 | 0.119 |
| Chloride | 107.14 (5.18) | 106.72 (4.47) | <0.001 | 0.087 |
| PO_2_ | 340.99 (114.67) | 339.91 (110.68) | 0.674 | 0.01 |
| PCO_2_ | 41.60 (6.60) | 41.49 (6.01) | 0.433 | 0.018 |
| PH | 7.39 (0.06) | 7.39 (0.05) | 0.26 | 0.026 |
| TotalCO_2_ | 25.98 (3.21) | 25.70 (2.57) | <0.001 | 0.096 |
| HR | 80.35 (12.23) | 79.23 (11.94) | <0.001 | 0.092 |
| SBP | 113.30 (19.59) | 115.68 (18.19) | <0.001 | 0.126 |
| DBP | 58.79 (12.18) | 60.05 (11.40) | <0.001 | 0.107 |
| MBP | 77.39 (14.61) | 78.76 (14.74) | <0.001 | 0.093 |
| CVP | 11.75 (20.31) | 11.11 (22.84) | 0.182 | 0.03 |
| RR | 16.05 (4.32) | 16.02 (4.12) | 0.793 | 0.006 |
| Temperature | 36.20 (0.75) | 36.25 (0.68) | 0.004 | 0.065 |
| SpO_2_ | 98.76 (2.95) | 98.95 (2.19) | 0.001 | 0.076 |
| Lactate | 1.55 (1.12) | 1.49 (0.66) | 0.003 | 0.07 |
| Urineoutput | 1793.66 (980.32) | 1893.17 (864.99) | <0.001 | 0.108 |
| MI (%) |  |  | <0.001 | 0.107 |
| No | 3704 (71.0) | 2081 (66.0) |  |  |
| Yes | 1516 (29.0) | 1073 (34.0) |  |  |
| Hypertension (%) |  |  | <0.001 | 0.12 |
| No | 1135 (21.7) | 536 (17.0) |  |  |
| Yes | 4085 (78.3) | 2618 (83.0) |  |  |
| AF (%) |  |  | <0.001 | 0.143 |
| No | 2682 (51.4) | 1845 (58.5) |  |  |
| Yes | 2538 (48.6) | 1309 (41.5) |  |  |
| Chronic_pulmonary_disease (%) | |  | <0.001 | 0.114 |
| No | 3972 (76.1) | 2547 (80.8) |  |  |
| Yes | 1248 (23.9) | 607 (19.2) |  |  |
| Chronic_renal_disease (%) | |  | <0.001 | 0.118 |
| No | 4033 (77.3) | 2586 (82.0) |  |  |
| Yes | 1187 (22.7) | 568 (18.0) |  |  |
| Chronic_liver_disease (%) | |  | <0.001 | 0.1 |
| No | 4977 (95.3) | 3043 (96.5) |  |  |
| Yes | 243 (4.7) | 111 (3.5) |  |  |
| Diabetes (%) |  |  | 0.89 | 0.005 |
| No | 3486 (66.8) | 1956 (62.0) |  |  |
| Yes | 1734 (33.2) | 1198 (38.0) |  |  |
| Malignant_cancer (%) | |  | 0.299 | 0.025 |
| No | 5077 (97.3) | 3070 (97.3) |  |  |
| Yes | 143 (2.7) | 84 (2.7) |  |  |
| Rheumatic_disease (%) | |  | 0.234 | 0.038 |
| No | 5052 (96.8) | 3066 (97.2) |  |  |
| Yes | 168 (3.2) | 88 (2.8) |  |  |
| Charlson_comorbidity_index | 4.82 (2.43) | 4.41 (2.31) | <0.001 | 0.172 |
| SOFA | 5.62 (2.98) | 4.70 (2.37) | <0.001 | 0.344 |
| Apsiii | 40.32 (18.90) | 36.58 (17.07) | <0.001 | 0.208 |
| Oasis | 32.24 (7.48) | 30.58 (7.62) | <0.001 | 0.22 |
| Vasoactive_drugs (%) | |  | <0.001 | 0.444 |
| No | 1467 (28.1) | 1552 (49.2) |  |  |
| Yes | 3753 (71.9) | 1602 (50.8) |  |  |
| Antibiotic (%) |  |  | <0.001 | 0.113 |
| No | 371 (7.1) | 324 (10.3) |  |  |
| Yes | 4849 (92.9) | 2830 (89.7) |  |  |
| ACEI (%) |  |  | <0.001 | 0.094 |
| No | 5062 (97.0) | 3001 (95.1) |  |  |
| Yes | 158 (3.0) | 153 (4.9) |  |  |
| Glucocorticoid (%) |  |  | 0.009 | 0.061 |
| No | 4982 (95.4) | 3048 (96.6) |  |  |
| Yes | 238 (4.6) | 106 (3.4) |  |  |
| Anticoagulant (%) |  |  | 0.478 | 0.017 |
| No | 4067 (77.9) | 2479 (78.6) |  |  |
| Yes | 1153 (22.1) | 675 (21.4) |  |  |
| Diuretics (%) |  |  | <0.001 | 0.709 |
| No | 2882 (55.2) | 714 (22.6) |  |  |
| Yes | 2338 (44.8) | 2440 (77.4) |  |  |
| RRT (%) |  |  | 0.057 | 0.046 |
| No | 5148 (98.6) | 3126 (99.1) |  |  |
| Yes | 72 (1.4) | 28 (0.9) |  |  |
| Mechanical_ventilation (%) | |  | <0.001 | 0.31 |
| No | 1292 (24.8) | 1231 (39.0) |  |  |
| Yes | 3928 (75.2) | 1923 (61.0) |  |  |
| Sepsis (%) |  |  | <0.001 | 0.909 |
| No | 2559 (49.0) | 2761 (87.5) |  |  |
| Yes | 2661 (51.0) | 393 (12.5) |  |  |
| Hospital_death (%) |  |  | <0.001 | 0.141 |
| No | 5064 (97.0) | 3122 (99.0) |  |  |
| Yes | 156 (3.0) | 32 (1.0) |  |  |
| 28day_death (%) |  |  | <0.001 | 0.126 |
| No | 5058 (96.9) | 3114 (98.7) |  |  |
| Yes | 162 (3.1) | 40 (1.3) |  |  |
| 365day_death (%) |  |  | <0.001 | 0.154 |
| No | 4756 (91.1) | 2997 (95.0) |  |  |
| Yes | 464 (8.9) | 157 (5.0) |  |  |
| Los_hospital | 10.70 (9.29) | 8.60 (6.23) | <0.001 | 0.266 |
| Los_icu | 3.92 (5.43) | 2.31 (3.36) | <0.001 | 0.355 |

Continuous variables expressed as mean ± standard deviation (SD).BMI: body mass index; WBC: white blood cell; RBC: red blood cell; RDW: red cell distribution width; INR: international normalized ratio; PT: prothrombin time; PTT: partial thromboplastin time; BUN: blood urea nitrogen; PO_2_: oxygen partial pressure; PCO_2_: carbon dioxide partial pressure; Total CO_2_: total carbon dioxide; HR: heart rate; SBP: systolic blood pressure; DBP: diastolic blood pressure; MBP: mean blood pressure; RR: respiratory rate; SpO2: percutaneous arterial oxygen saturation; SOFA: Sequential organ failure score; Apsiii: Simplified Acute Physiology Score III; Oasis: Oxford acute severity of illness score; MI: myocardial infarction; AF: atrial fibrillation; RRT: renal replacement therapy; Los: length of stay.

Supplementary Table 2. Comparison of baseline characteristics after PSM

| Variables | Control (N = 2507) | β-blocker (N = 2507) | *P* | SMD |
| --- | --- | --- | --- | --- |
| Gender (%) |  |  | 0.172 | 0.039 |
| Female | 756 (30.2) | 711 (28.4) |  |  |
| Male | 1751 (69.8) | 1796 (71.6) |  |  |
| Admission_type (%) |  |  | 0.797 | 0.008 |
| Emergency | 1071 (42.7) | 1061 (42.3) |  |  |
| Floor | 1436 (57.3) | 1446 (57.7) |  |  |
| Race (%) |  |  | 0.797 | 0.019 |
| Black | 100 (4.0) | 107 (4.3) |  |  |
| Other | 609 (24.3) | 621 (24.8) |  |  |
| White | 1798 (71.7) | 1779 (71.0) |  |  |
| Admission_age | 69.23 (12.11) | 68.71 (11.17) | 0.118 | 0.044 |
| BMI | 29.79 (6.14) | 29.83 (5.87) | 0.797 | 0.007 |
| WBC | 11.70 (5.41) | 11.65 (5.13) | 0.734 | 0.01 |
| RBC | 3.33 (0.72) | 3.33 (0.68) | 0.713 | 0.01 |
| Platelet | 164.62 (66.87) | 163.08 (68.33) | 0.419 | 0.023 |
| Hemoglobin | 10.01 (2.12) | 10.03 (2.01) | 0.67 | 0.012 |
| RDW | 13.92 (1.63) | 13.81 (1.64) | 0.016 | 0.068 |
| Hematocrit | 30.21 (6.22) | 30.25 (5.81) | 0.813 | 0.007 |
| INR | 1.40 (0.29) | 1.40 (0.32) | 0.471 | 0.02 |
| PT | 15.29 (3.03) | 15.35 (3.48) | 0.549 | 0.017 |
| PTT | 40.29 (24.12) | 39.92 (24.45) | 0.595 | 0.015 |
| Aniongap | 11.71 (3.26) | 11.55 (3.18) | 0.078 | 0.05 |
| Bicarbonate | 23.21 (2.82) | 23.23 (2.75) | 0.792 | 0.007 |
| BUN | 19.33 (11.47) | 18.89 (10.57) | 0.153 | 0.04 |
| Creatinine | 1.07 (0.94) | 1.07 (0.94) | 0.934 | 0.002 |
| Glucose | 127.47 (41.83) | 126.80 (55.61) | 0.63 | 0.014 |
| Sodium | 138.29 (3.27) | 138.18 (2.98) | 0.208 | 0.036 |
| Potassium | 4.32 (0.57) | 4.32 (0.52) | 0.879 | 0.004 |
| Magnesium | 2.35 (0.54) | 2.35 (0.52) | 0.981 | 0.001 |
| Chloride | 106.76 (5.15) | 106.73 (4.53) | 0.835 | 0.006 |
| PO_2_ | 336.79 (116.90) | 337.43 (112.43) | 0.843 | 0.006 |
| PCO_2_ | 41.47 (6.43) | 41.52 (6.23) | 0.798 | 0.007 |
| PH | 7.39 (0.06) | 7.39 (0.05) | 0.622 | 0.014 |
| TotalCO_2_ | 25.82 (3.05) | 25.77 (2.66) | 0.567 | 0.016 |
| HR | 79.77 (12.37) | 79.41 (12.20) | 0.295 | 0.03 |
| SBP | 115.54 (20.12) | 115.46 (18.37) | 0.887 | 0.004 |
| DBP | 59.73 (12.36) | 59.71 (11.42) | 0.946 | 0.002 |
| MBP | 78.73 (15.32) | 78.57 (14.87) | 0.705 | 0.011 |
| CVP | 11.64 (21.22) | 11.56 (24.23) | 0.898 | 0.004 |
| RR | 16.12 (4.35) | 16.09 (4.25) | 0.798 | 0.007 |
| Temperature | 36.23 (0.73) | 36.24 (0.71) | 0.688 | 0.011 |
| SpO_2_ | 98.83 (2.38) | 98.87 (2.28) | 0.565 | 0.016 |
| Lactate | 1.49 (0.80) | 1.50 (0.69) | 0.944 | 0.002 |
| Urineoutput | 1868.81 (989.75) | 1869.35 (869.00) | 0.984 | 0.001 |
| MI (%) |  |  | 0.131 | 0.043 |
| No | 1721 (68.6) | 1670 (66.6) |  |  |
| Yes | 786 (31.4) | 837 (33.4) |  |  |
| Hypertension (%) |  |  | 0.855 | 0.006 |
| No | 460 (18.3) | 454 (18.1) |  |  |
| Yes | 2047 (81.7) | 2053 (81.9) |  |  |
| AF (%) |  |  | 0.443 | 0.022 |
| No | 1384 (55.2) | 1412 (56.3) |  |  |
| Yes | 1123 (44.8) | 1095 (43.7) |  |  |
| Chronic_pulmonary_disease (%) | |  | 0.677 | 0.013 |
| No | 1976 (78.8) | 1989 (79.3) |  |  |
| Yes | 531 (21.2) | 518 (20.7) |  |  |
| Chronic_renal_disease (%) | |  | 0.722 | 0.011 |
| No | 2011 (80.2) | 2022 (80.7) |  |  |
| Yes | 496 (19.8) | 485 (19.3) |  |  |
| Chronic_liver_disease (%) | |  | 1 | <0.001 |
| No | 2416 (96.4) | 2416 (96.4) |  |  |
| Yes | 91 (3.6) | 91 (3.6) |  |  |
| Diabetes (%) |  |  | 0.953 | 0.002 |
| No | 1588 (63.3) | 1585 (63.2) |  |  |
| Yes | 919 (36.7) | 922 (36.8) |  |  |
| Malignant_cancer (%) | |  | 0.739 | 0.012 |
| No | 2430 (96.9) | 2435 (97.1) |  |  |
| Yes | 77 (3.1) | 72 (2.9) |  |  |
| Rheumatic_disease (%) | |  | 0.524 | 0.02 |
| No | 2421 (96.6) | 2430 (96.9) |  |  |
| Yes | 86 (3.4) | 77 (3.1) |  |  |
| Charlson_comorbidity_index | 4.64 (2.39) | 4.57 (2.32) | 0.233 | 0.034 |
| SOFA | 4.98 (2.64) | 4.84 (2.45) | 0.052 | 0.055 |
| Apsiii | 38.08 (17.43) | 37.37 (17.42) | 0.148 | 0.041 |
| Oasis | 31.47 (7.56) | 31.01 (7.56) | 0.031 | 0.061 |
| Vasoactive_drugs (%) | |  | 0.096 | 0.048 |
| No | 999 (39.8) | 1058 (42.2) |  |  |
| Yes | 1508 (60.2) | 1449 (57.8) |  |  |
| Antibiotic (%) |  |  | 0.582 | 0.017 |
| No | 259 (10.3) | 272 (10.8) |  |  |
| Yes | 2248 (89.7) | 2235 (89.2) |  |  |
| ACEI (%) |  |  | 0.73 | 0.012 |
| No | 2400 (95.7) | 2394 (95.5) |  |  |
| Yes | 107 (4.3) | 113 (4.5) |  |  |
| Glucocorticoid (%) |  |  | 0.769 | 0.01 |
| No | 2408 (96.1) | 2413 (96.3) |  |  |
| Yes | 99 (3.9) | 94 (3.7) |  |  |
| Anticoagulant (%) |  |  | 0.372 | 0.026 |
| No | 1890 (75.4) | 1918 (76.5) |  |  |
| Yes | 617 (24.6) | 589 (23.5) |  |  |
| Diuretics (%) |  |  | 0.142 | 0.042 |
| No | 743 (29.6) | 695 (27.7) |  |  |
| Yes | 1764 (70.4) | 1812 (72.3) |  |  |
| RRT (%) |  |  | 0.769 | 0.012 |
| No | 2485 (99.1) | 2482 (99.0) |  |  |
| Yes | 22 (0.9) | 25 (1.0) |  |  |
| Mechanical_ventilation (%) | |  | 0.018 | 0.068 |
| No | 829 (33.1) | 910 (36.3) |  |  |
| Yes | 1678 (66.9) | 1597 (63.7) |  |  |
| Sepsis (%) |  |  | <0.001 | 0.705 |
| No | 1384 (55.2) | 2145 (85.6) |  |  |
| Yes | 1123 (44.8) | 362 (14.4) |  |  |
| Hospital_death (%) |  |  | 0.004 | 0.084 |
| No | 2452 (97.8) | 2479 (98.9) |  |  |
| Yes | 55 (2.2) | 28 (1.1) |  |  |
| 28day_death (%) |  |  | 0.006 | 0.08 |
| No | 2443 (97.4) | 2471 (98.6) |  |  |
| Yes | 64 (2.6) | 36 (1.4) |  |  |
| 365day_death (%) |  |  | 0.009 | 0.076 |
| No | 2320 (92.5) | 2367 (94.4) |  |  |
| Yes | 187 (7.5) | 140 (5.6) |  |  |
| Los_hospital | 9.81 (8.48) | 8.79 (6.65) | <0.001 | 0.134 |
| Los_icu | 3.25 (3.86) | 2.46 (3.70) | <0.001 | 0.209 |

Continuous variables expressed as mean ± standard deviation (SD).PSM: propensity score matching; BMI: body mass index; WBC: white blood cell; RBC: red blood cell; RDW: red cell distribution width; INR: international normalized ratio; PT: prothrombin time; PTT: partial thromboplastin time; BUN: blood urea nitrogen; PO2: oxygen partial pressure; PCO2: carbon dioxide partial pressure; Total CO2: total carbon dioxide; HR: heart rate; SBP: systolic blood pressure; DBP: diastolic blood pressure; MBP: mean blood pressure; RR: respiratory rate; SpO2: percutaneous arterial oxygen saturation; SOFA: Sequential organ failure score; Apsiii: Simplified Acute Physiology Score III; Oasis: Oxford acute severity of illness score; MI: myocardial infarction; AF: atrial fibrillation; RRT: renal replacement therapy; Los: length of stay.

Supplementary Table 3. Comparison of baseline characteristics after IPTW

| Variables | Control (N = 8289.2) | β-blocker (N = 7116.2) | *P* | SMD |
| --- | --- | --- | --- | --- |
| Gender (%) |  |  | 0.609 | 0.013 |
| Female | 2526.1 (30.5) | 2126.7 (29.9) |  |  |
| Male | 5763.1 (69.5) | 4989.5 (70.1) |  |  |
| Admission_type (%) |  |  | 0.888 | 0.003 |
| Emergency | 3583.0 (43.2) | 3088.2 (43.4) |  |  |
| Floor | 4706.3 (56.8) | 4028.0 (56.6) |  |  |
| Race (%) |  |  | 0.593 | 0.025 |
| Black | 345.4 (4.2) | 309.4 (4.3) |  |  |
| Other | 1966.1 (23.7) | 1756.6 (24.7) |  |  |
| White | 5977.7 (72.1) | 5050.2 (71.0) |  |  |
| Admission_age | 69.21 (12.18) | 69.00 (11.29) | 0.473 | 0.018 |
| BMI | 29.62 (6.14) | 29.70 (5.85) | 0.558 | 0.014 |
| WBC | 12.00 (6.37) | 11.67 (5.25) | 0.018 | 0.056 |
| RBC | 3.30 (0.71) | 3.33 (0.69) | 0.09 | 0.042 |
| Platelet | 163.21 (67.45) | 164.39 (69.73) | 0.501 | 0.017 |
| Hemoglobin | 9.93 (2.10) | 10.02 (2.03) | 0.105 | 0.04 |
| RDW | 14.02 (1.72) | 13.91 (1.76) | 0.014 | 0.064 |
| Hematocrit | 30.00 (6.17) | 30.22 (5.89) | 0.148 | 0.036 |
| INR | 1.43 (0.41) | 1.41 (0.36) | 0.011 | 0.065 |
| PT | 15.72 (4.94) | 15.41 (3.91) | 0.005 | 0.069 |
| PTT | 40.87 (24.77) | 40.31 (24.90) | 0.372 | 0.023 |
| Aniongap | 11.79 (3.36) | 11.70 (3.25) | 0.247 | 0.029 |
| Bicarbonate | 23.19 (2.92) | 23.23 (2.82) | 0.551 | 0.015 |
| BUN | 19.97 (12.37) | 19.48 (11.43) | 0.111 | 0.041 |
| Creatinine | 1.14 (1.10) | 1.11 (1.01) | 0.293 | 0.028 |
| Glucose | 127.30 (41.58) | 127.39 (64.28) | 0.957 | 0.002 |
| Sodium | 138.45 (3.33) | 138.25 (3.00) | 0.013 | 0.061 |
| Potassium | 4.31 (0.57) | 4.32 (0.53) | 0.57 | 0.014 |
| Magnesium | 2.38 (0.55) | 2.35 (0.52) | 0.043 | 0.05 |
| Chloride | 106.88 (5.17) | 106.70 (4.65) | 0.143 | 0.037 |
| PO_2_ | 338.05 (116.94) | 336.90 (112.40) | 0.69 | 0.01 |
| PCO_2_ | 41.61 (6.52) | 41.60 (6.37) | 0.922 | 0.003 |
| PH | 7.39 (0.06) | 7.39 (0.06) | 0.835 | 0.005 |
| TotalCO_2_ | 25.89 (3.13) | 25.84 (2.70) | 0.483 | 0.017 |
| HR | 79.94 (12.23) | 79.70 (12.30) | 0.446 | 0.019 |
| SBP | 114.69 (20.24) | 115.55 (18.72) | 0.081 | 0.044 |
| DBP | 59.41 (12.49) | 59.79 (11.57) | 0.209 | 0.032 |
| MBP | 78.16 (14.79) | 78.65 (15.17) | 0.2 | 0.033 |
| CVP | 11.69 (21.34) | 11.50 (23.86) | 0.748 | 0.008 |
| RR | 16.08 (4.34) | 16.08 (4.27) | 0.961 | 0.001 |
| Temperature | 36.22 (0.74) | 36.23 (0.73) | 0.591 | 0.014 |
| SpO_2_ | 98.78 (2.75) | 98.84 (2.39) | 0.414 | 0.02 |
| Lactate | 1.53 (1.01) | 1.50 (0.73) | 0.129 | 0.036 |
| Urineoutput | 1827.18 (977.93) | 1861.86 (897.96) | 0.141 | 0.037 |
| MI (%) |  |  | 0.271 | 0.027 |
| No | 5731.4 (69.1) | 4830.4 (67.9) |  |  |
| Yes | 2557.8 (30.9) | 2285.9 (32.1) |  |  |
| Hypertension (%) |  |  | 0.11 | 0.04 |
| No | 1671.2 (20.2) | 1322.1 (18.6) |  |  |
| Yes | 6618.0 (79.8) | 5794.1 (81.4) |  |  |
| AF (%) |  |  | 0.117 | 0.039 |
| No | 4445.6 (53.6) | 3954.5 (55.6) |  |  |
| Yes | 3843.6 (46.4) | 3161.8 (44.4) |  |  |
| Chronic_pulmonary_disease (%) | |  | 0.29 | 0.026 |
| No | 6432.5 (77.6) | 5599.9 (78.7) |  |  |
| Yes | 1856.7 (22.4) | 1516.3 (21.3) |  |  |
| Chronic_renal_disease (%) | |  | 0.312 | 0.026 |
| No | 6512.0 (78.6) | 5664.4 (79.6) |  |  |
| Yes | 1777.2 (21.4) | 1451.9 (20.4) |  |  |
| Chronic_liver_disease (%) | |  | 0.667 | 0.011 |
| No | 7937.1 (95.8) | 6829.5 (96.0) |  |  |
| Yes | 352.2 (4.2) | 286.7 (4.0) |  |  |
| Diabetes (%) |  |  | 0.299 | 0.026 |
| No | 5414.7 (65.3) | 4561.3 (64.1) |  |  |
| Yes | 2874.5 (34.7) | 2554.9 (35.9) |  |  |
| Malignant_cancer (%) | |  | 0.788 | 0.007 |
| No | 8054.1 (97.2) | 6906.1 (97.0) |  |  |
| Yes | 235.2 (2.8) | 210.2 (3.0) |  |  |
| Rheumatic_disease (%) | |  | 0.997 | <0.001 |
| No | 8028.4 (96.9) | 6892.2 (96.9) |  |  |
| Yes | 260.8 (3.1) | 224.1 (3.1) |  |  |
| Charlson_comorbidity_index | 4.72 (2.41) | 4.64 (2.36) | 0.176 | 0.034 |
| SOFA | 5.27 (2.87) | 4.98 (2.55) | <0.001 | 0.109 |
| Apsiii | 39.17 (18.21) | 38.17 (17.86) | 0.025 | 0.056 |
| Oasis | 31.70 (7.52) | 31.37 (7.60) | 0.075 | 0.044 |
| Vasoactive_drugs (%) | |  | <0.001 | 0.109 |
| No | 2949.5 (35.6) | 2907.0 (40.9) |  |  |
| Yes | 5339.7 (64.4) | 4209.2 (59.1) |  |  |
| Antibiotic (%) |  |  | 0.114 | 0.041 |
| No | 768.4 (9.3) | 747.6 (10.5) |  |  |
| Yes | 7520.9 (90.7) | 6368.7 (89.5) |  |  |
| ACEI (%) |  |  | 0.301 | 0.027 |
| No | 7967.0 (96.1) | 6802.0 (95.6) |  |  |
| Yes | 322.3 (3.9) | 314.3 (4.4) |  |  |
| Glucocorticoid (%) |  |  | 0.711 | 0.01 |
| No | 7934.6 (95.7) | 6825.4 (95.9) |  |  |
| Yes | 354.7 (4.3) | 290.8 (4.1) |  |  |
| Anticoagulant (%) |  |  | 0.509 | 0.017 |
| No | 6347.9 (76.6) | 5398.5 (75.9) |  |  |
| Yes | 1941.4 (23.4) | 1717.8 (24.1) |  |  |
| Diuretics (%) |  |  | <0.001 | 0.169 |
| No | 3612.9 (43.6) | 2517.0 (35.4) |  |  |
| Yes | 4676.3 (56.4) | 4599.2 (64.6) |  |  |
| RRT (%) |  |  | 0.731 | 0.009 |
| No | 8191.5 (98.8) | 7039.0 (98.9) |  |  |
| Yes | 97.8 (1.2) | 77.3 (1.1) |  |  |
| Mechanical_ventilation (%) | |  | 0.002 | 0.077 |
| No | 2511.4 (30.3) | 2411.2 (33.9) |  |  |
| Yes | 5777.8 (69.7) | 4705.1 (66.1) |  |  |
| Sepsis (%) |  |  | <0.001 | 0.712 |
| No | 4410.5 (53.2) | 5998.8 (84.3) |  |  |
| Yes | 3878.8 (46.8) | 1117.5 (15.7) |  |  |
| Hospital_death (%) |  |  | 0.001 | 0.091 |
| No | 8072.4 (97.4) | 7020.2 (98.7) |  |  |
| Yes | 216.9 (2.6) | 96.0 (1.3) |  |  |
| 28day_death (%) |  |  | 0.002 | 0.083 |
| No | 8059.3 (97.2) | 7004.8 (98.4) |  |  |
| Yes | 230.0 (2.8) | 111.4 (1.6) |  |  |
| 365day_death (%) |  |  | 0.008 | 0.07 |
| No | 7621.0 (91.9) | 6670.2 (93.7) |  |  |
| Yes | 668.3 (8.1) | 446.1 (6.3) |  |  |
| Los_hospital | 10.40 (9.16) | 9.00 (6.74) | <0.001 | 0.174 |
| Los_icu | 3.65 (4.95) | 2.56 (3.93) | <0.001 | 0.243 |

Continuous variables expressed as mean ± standard deviation (SD).IPTW: inverse probability of treatment weighting; BMI: body mass index; WBC: white blood cell; RBC: red blood cell; RDW: red cell distribution width; INR: international normalized ratio; PT: prothrombin time; PTT: partial thromboplastin time; BUN: blood urea nitrogen; PO2: oxygen partial pressure; PCO2: carbon dioxide partial pressure; Total CO2: total carbon dioxide; HR: heart rate; SBP: systolic blood pressure; DBP: diastolic blood pressure; MBP: mean blood pressure; RR: respiratory rate; SpO2: percutaneous arterial oxygen saturation; SOFA: Sequential organ failure score; Apsiii: Simplified Acute Physiology Score III; Oasis: Oxford acute severity of illness score; MI: myocardial infarction; AF: atrial fibrillation; RRT: renal replacement therapy; Los: length of stay.

Supplementary Table 4. Comparison of baseline characteristics after OW

| Variables | Control (N = 1529.7) | β-blocker (N = 1526.3) | *P* | SMD |
| --- | --- | --- | --- | --- |
| Gender (%) |  |  | 0.958 | 0.001 |
| Female | 455.8 (29.8) | 453.8 (29.7) |  |  |
| Male | 1073.9 (70.2) | 1072.4 (70.3) |  |  |
| Admission_type (%) |  |  | 0.992 | <0.001 |
| Emergency | 658.4 (43.0) | 657.1 (43.1) |  |  |
| Floor | 871.3 (57.0) | 869.2 (56.9) |  |  |
| Race (%) |  |  | 1 | <0.001 |
| Black | 65.4 (4.3) | 65.3 (4.3) |  |  |
| Other | 372.4 (24.3) | 371.7 (24.4) |  |  |
| White | 1091.9 (71.4) | 1089.3 (71.4) |  |  |
| Admission_age | 69.08 (12.10) | 69.07 (11.20) | 0.984 | 0.001 |
| BMI | 29.73 (6.15) | 29.73 (5.85) | 0.993 | <0.001 |
| WBC | 11.70 (5.61) | 11.69 (5.23) | 0.959 | 0.001 |
| RBC | 3.32 (0.71) | 3.32 (0.68) | 0.993 | <0.001 |
| Platelet | 163.93 (66.13) | 163.92 (69.36) | 0.997 | <0.001 |
| Hemoglobin | 10.01 (2.11) | 10.01 (2.02) | 0.986 | <0.001 |
| RDW | 13.90 (1.60) | 13.89 (1.73) | 0.9 | 0.003 |
| Hematocrit | 30.18 (6.17) | 30.19 (5.87) | 0.995 | <0.001 |
| INR | 1.41 (0.32) | 1.41 (0.35) | 0.864 | 0.005 |
| PT | 15.42 (3.51) | 15.40 (3.82) | 0.825 | 0.006 |
| PTT | 40.26 (24.09) | 40.23 (24.84) | 0.962 | 0.001 |
| Aniongap | 11.69 (3.30) | 11.68 (3.23) | 0.957 | 0.001 |
| Bicarbonate | 23.22 (2.87) | 23.22 (2.80) | 0.979 | 0.001 |
| BUN | 19.40 (11.65) | 19.37 (11.25) | 0.913 | 0.003 |
| Creatinine | 1.10 (1.03) | 1.10 (0.98) | 0.977 | 0.001 |
| Glucose | 127.26 (41.04) | 127.20 (62.63) | 0.97 | 0.001 |
| Sodium | 138.27 (3.30) | 138.27 (2.98) | 0.976 | 0.001 |
| Potassium | 4.32 (0.57) | 4.32 (0.52) | 0.995 | <0.001 |
| Magnesium | 2.36 (0.54) | 2.36 (0.52) | 0.987 | <0.001 |
| Chloride | 106.75 (5.12) | 106.75 (4.62) | 0.997 | <0.001 |
| PO_2_ | 337.13 (117.65) | 337.21 (112.01) | 0.98 | 0.001 |
| PCO_2_ | 41.58 (6.43) | 41.58 (6.34) | 0.986 | <0.001 |
| PH | 7.39 (0.06) | 7.39 (0.06) | 0.978 | 0.001 |
| TotalCO_2_ | 25.83 (3.05) | 25.83 (2.70) | 0.995 | <0.001 |
| HR | 79.61 (12.19) | 79.60 (12.29) | 0.971 | 0.001 |
| SBP | 115.44 (20.34) | 115.44 (18.71) | 0.999 | <0.001 |
| DBP | 59.71 (12.59) | 59.71 (11.57) | 0.996 | <0.001 |
| MBP | 78.57 (14.76) | 78.57 (15.21) | 0.998 | <0.001 |
| CVP | 11.52 (21.06) | 11.51 (23.96) | 0.982 | 0.001 |
| RR | 16.09 (4.32) | 16.09 (4.28) | 0.99 | <0.001 |
| Temperature | 36.23 (0.73) | 36.23 (0.72) | 0.998 | <0.001 |
| SpO_2_ | 98.84 (2.48) | 98.84 (2.36) | 0.958 | 0.001 |
| Lactate | 1.50 (0.84) | 1.50 (0.73) | 0.939 | 0.002 |
| Urineoutput | 1861.09 (973.86) | 1862.13 (889.57) | 0.965 | 0.001 |
| MI (%) |  |  | 0.974 | 0.001 |
| No | 1043.3 (68.2) | 1040.4 (68.2) |  |  |
| Yes | 486.4 (31.8) | 485.9 (31.8) |  |  |
| Hypertension (%) |  |  | 0.982 | 0.001 |
| No | 284.6 (18.6) | 283.7 (18.6) |  |  |
| Yes | 1245.0 (81.4) | 1242.6 (81.4) |  |  |
| AF (%) |  |  | 0.965 | 0.001 |
| No | 846.4 (55.3) | 845.4 (55.4) |  |  |
| Yes | 683.3 (44.7) | 680.9 (44.6) |  |  |
| Chronic_pulmonary_disease (%) | |  | 0.981 | 0.001 |
| No | 1201.8 (78.6) | 1199.5 (78.6) |  |  |
| Yes | 327.9 (21.4) | 326.8 (21.4) |  |  |
| Chronic_renal_disease (%) | |  | 0.975 | 0.001 |
| No | 1218.7 (79.7) | 1216.5 (79.7) |  |  |
| Yes | 310.9 (20.3) | 309.8 (20.3) |  |  |
| Chronic_liver_disease (%) | |  | 0.963 | 0.001 |
| No | 1469.3 (96.1) | 1466.4 (96.1) |  |  |
| Yes | 60.4 (3.9) | 59.9 (3.9) |  |  |
| Diabetes (%) |  |  | 0.985 | <0.001 |
| No | 980.9 (64.1) | 978.4 (64.1) |  |  |
| Yes | 548.8 (35.9) | 547.9 (35.9) |  |  |
| Malignant_cancer (%) | |  | 0.996 | <0.001 |
| No | 1485.7 (97.1) | 1482.4 (97.1) |  |  |
| Yes | 44.0 (2.9) | 43.9 (2.9) |  |  |
| Rheumatic_disease (%) | |  | 0.991 | <0.001 |
| No | 1481.3 (96.8) | 1478.0 (96.8) |  |  |
| Yes | 48.4 (3.2) | 48.2 (3.2) |  |  |
| Charlson_comorbidity_index | 4.64 (2.39) | 4.64 (2.35) | 0.958 | 0.001 |
| SOFA | 4.98 (2.68) | 4.97 (2.52) | 0.913 | 0.003 |
| Apsiii | 38.06 (17.42) | 38.02 (17.77) | 0.937 | 0.002 |
| Oasis | 31.37 (7.50) | 31.36 (7.58) | 0.965 | 0.001 |
| Vasoactive_drugs (%) | |  | 0.954 | 0.001 |
| No | 611.4 (40.0) | 611.2 (40.0) |  |  |
| Yes | 918.2 (60.0) | 915.1 (60.0) |  |  |
| Antibiotic (%) |  |  | 0.99 | <0.001 |
| No | 159.1 (10.4) | 158.9 (10.4) |  |  |
| Yes | 1370.6 (89.6) | 1367.4 (89.6) |  |  |
| ACEI (%) |  |  | 0.992 | <0.001 |
| No | 1463.6 (95.7) | 1460.3 (95.7) |  |  |
| Yes | 66.0 (4.3) | 66.0 (4.3) |  |  |
| Glucocorticoid (%) |  |  | 0.99 | <0.001 |
| No | 1468.1 (96.0) | 1464.9 (96.0) |  |  |
| Yes | 61.6 (4.0) | 61.4 (4.0) |  |  |
| Anticoagulant (%) |  |  | 0.973 | 0.001 |
| No | 1163.3 (76.1) | 1161.3 (76.1) |  |  |
| Yes | 366.4 (23.9) | 365.0 (23.9) |  |  |
| Diuretics (%) |  |  | 0.917 | 0.003 |
| No | 511.0 (33.4) | 507.9 (33.3) |  |  |
| Yes | 1018.7 (66.6) | 1018.4 (66.7) |  |  |
| RRT (%) |  |  | 0.998 | <0.001 |
| No | 1513.8 (99.0) | 1510.5 (99.0) |  |  |
| Yes | 15.8 (1.0) | 15.8 (1.0) |  |  |
| Mechanical_ventilation (%) | |  | 0.97 | 0.001 |
| No | 510.3 (33.4) | 509.8 (33.4) |  |  |
| Yes | 1019.4 (66.6) | 1016.4 (66.6) |  |  |
| Sepsis (%) |  |  | <0.001 | 0.667 |
| No | 852.1 (55.7) | 1292.2 (84.7) |  |  |
| Yes | 677.6 (44.3) | 234.1 (15.3) |  |  |
| Hospital_death (%) |  |  | 0.015 | 0.067 |
| No | 1496.2 (97.8) | 1506.3 (98.7) |  |  |
| Yes | 33.4 (2.2) | 19.9 (1.3) |  |  |
| 28day_death (%) |  |  | 0.02 | 0.063 |
| No | 1492.8 (97.6) | 1502.9 (98.5) |  |  |
| Yes | 36.9 (2.4) | 23.4 (1.5) |  |  |
| 365day_death (%) |  |  | 0.06 | 0.05 |
| No | 1417.3 (92.7) | 1433.2 (93.9) |  |  |
| Yes | 112.4 (7.3) | 93.1 (6.1) |  |  |
| Los_hospital | 10.07 (8.84) | 8.97 (6.76) | <0.001 | 0.14 |
| Los_icu | 3.38 (4.34) | 2.54 (3.86) | <0.001 | 0.205 |

Continuous variables expressed as mean ± standard deviation (SD).OW: overlap weighting; BMI: body mass index; WBC: white blood cell; RBC: red blood cell; RDW: red cell distribution width; INR: international normalized ratio; PT: prothrombin time; PTT: partial thromboplastin time; BUN: blood urea nitrogen; PO2: oxygen partial pressure; PCO2: carbon dioxide partial pressure; Total CO2: total carbon dioxide; HR: heart rate; SBP: systolic blood pressure; DBP: diastolic blood pressure; MBP: mean blood pressure; RR: respiratory rate; SpO2: percutaneous arterial oxygen saturation; SOFA: Sequential organ failure score; Apsiii: Simplified Acute Physiology Score III; Oasis: Oxford acute severity of illness score; MI: myocardial infarction; AF: atrial fibrillation; RRT: renal replacement therapy; Los: length of stay.

## Supplementary Figures


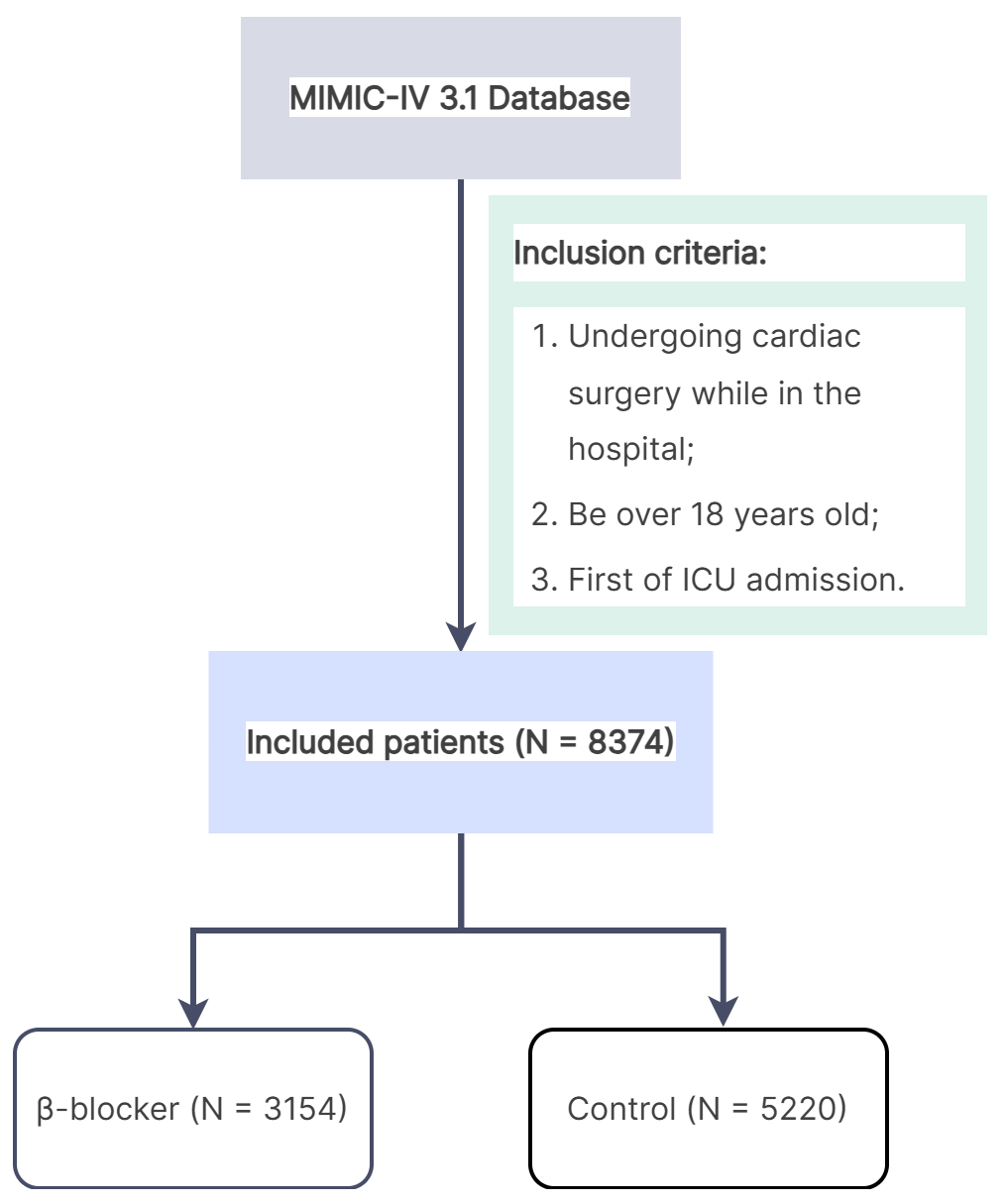


Supplementary Figure 1 Flow chart of drainage

Supplementary Figure 2 Proportion of Missing Variables

BMI: body mass index; WBC: white blood cell; RBC: red blood cell; RDW: red cell distribution width; INR: international normalized ratio; PT: prothrombin time; PTT: partial thromboplastin time; BUN: blood urea nitrogen; PO2: oxygen partial pressure; PCO2: carbon dioxide partial pressure; Total CO2: total carbon dioxide; HR: heart rate; SBP: systolic blood pressure; DBP: diastolic blood pressure; MBP: mean blood pressure; RR: respiratory rate; SpO2: percutaneous arterial oxygen saturation; SOFA: Sequential organ failure score; Apsiii: Simplified Acute Physiology Score III; Oasis: Oxford acute severity of illness score.


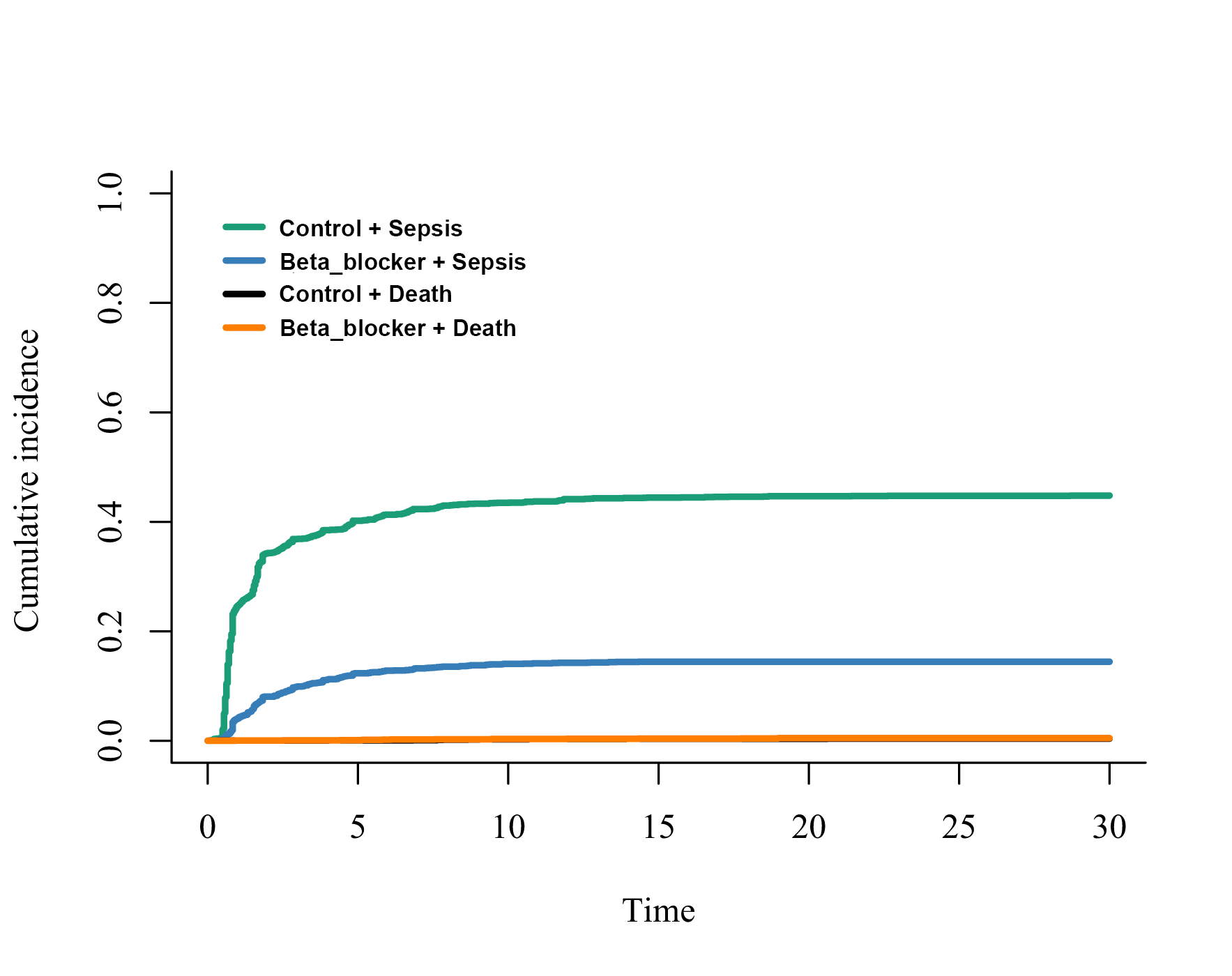


Supplementary Figure 3 Cumulative incidence functions for sepsis and in-hospital death in β-blocker and control groups after cardiac surgery.


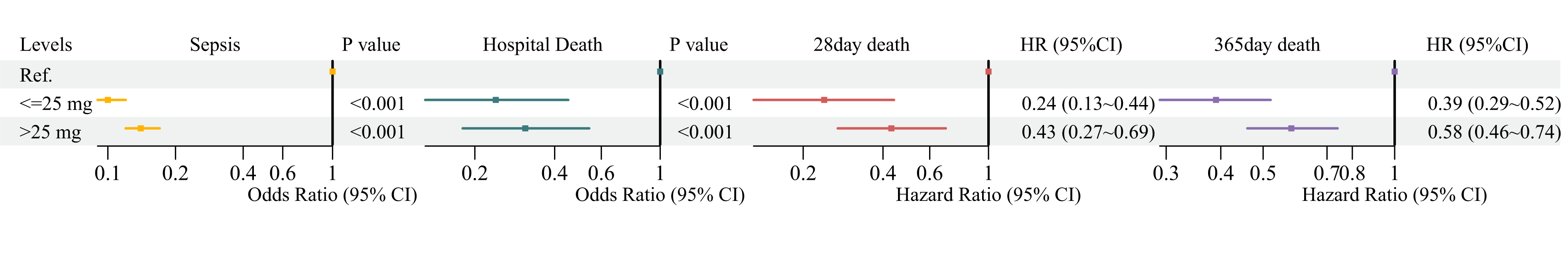


Supplementary Figure 4 Dose-response subgroup analysis of metoprolol associations with sepsis incidence, in-hospital mortality, and both 28-day and 1-year postoperative mortality following cardiac surgery.
